# Insulin autoimmune syndrome induced by exogenous insulin injection: a four-case series

**DOI:** 10.1186/s12902-019-0482-0

**Published:** 2019-12-28

**Authors:** Yimin Shen, Xiaoxiao Song, Yuezhong Ren

**Affiliations:** 1 Department of Endocrinology, The Second Affiliated Hospital of Zhejiang University School of Medicine, Zhejiang, 310009 China; 2 Zhejiang University School of Medicine, Zhejiang, 310058 China

**Keywords:** Insulin autoimmune syndrome, Hyperinsulinism, Hypoglycemia, Glucose, Diabetes, Acarbose, Steroid

## Abstract

**Background:**

Insulin autoimmune syndrome (IAS) is a rare cause of hypoglycemia and is characterized by the presence of insulin autoantibodies. Patients with IAS usually complain of hypoglycemia without any previous insulin received. Glucocorticoids and immunosuppressants are used to treat IAS.

**Case presentation:**

We report four patients with diabetes who were diagnosed with non-classical IAS and describe the treatment of these patients. Moreover, the differential diagnosis with hyperinsulinism is discussed.

**Conclusion:**

High levels of insulin autoantibodies, as well as hyperinsulinemic hypoglycemia, are found in patients with diabetes mellitus and prior exogenous insulin exposure. This situation that we classified as non-classical IAS should be attached importance to.

## Background

Classical insulin autoimmune syndrome (IAS) is characterized by extremely high serum insulin concentrations, as well as spontaneous hypoglycemia [1]. In IAS, there are also high titers of autoantibodies against endogenous insulin, which occur without any prior exposure to exogenous insulin [2, 3]. Hypoglycemia that results from exogenous insulin administration can also manifest with symptoms resembling those of IAS. Patients usually present with postprandial hypoglycemia, marked neuroglycopenic symptoms of confusion, and an altered state of consciousness in these two conditions [1]. In this case series, we introduce a new concept of non-classical IAS and elaborate on insulin-resistant syndrome, as well as relationships with other hyperinsulinemic diseases.

## Research methods

All the measurements of hormone were made in the Laboratory of The Second Affiliated Hospital of Zhejiang University. Serum insulin concentrations were measured by automated microparticle enzyme immunoassays (Abbott AxSYM MEIA; Abbott Laboratories, Abbott Park, IL, USA). C-peptide concentrations were measured by a specific immunoradiometric assay (Immunotech, Prague, Czech Republic). The qualitatively assessment of circulating immune complexes were precipitation with polyethylene glycol followed by an insulin assay of the supernatant [2]. The reduced accuracy of insulin measurement is not clinically relevant in patients with IAS because of the high serum insulin values. Moreover, there is no cross-reactivity of the insulin assay with synthetic insulin in this detection method.

## Case presentations

### Case 1

This patient was a 79-year-old man who had a 10-year history of hyperglycemia in addition to fluctuating glucose concentrations for more than 1 year. This was accompanied by diabetic nephropathy as well as a history of gout for more than 30 years. His glucose concentrations were initially well controlled with metformin after diagnosis of type 2 diabetes mellitus. A subcutaneous insulin pump (lispro) was recently added to his treatment regimen for renal insufficiency, and metformin was gradually changed to Humulin R (6–4-4 units, three times a day) combined with glargine 12 units a day. Thereafter, he had recurrent midnight hypoglycemia and daytime hyperglycemia, with blood glucose concentrations up to 20 mmol/L. Therefore, he was treated with acarbose (50 mg three times a day) instead of insulin injections. At this time, his C-peptide values were 5.45/6.94/6.94/7.89/7.17 nmol/L at 0/0.5/1/2/3 h, respectively (normal range: 0.27–1.28 nmol/L). His insulin concentrations were > 1000.00/> 1000.00 pmol/L at 0/2 h, respectively (normal range: 13–161 pmol/L), during an oral glucose tolerance test (OGTT), his HbA1c concentration was 10.2%, and an anti-insulin antibody test was positive (reference level: < 0.4). His glucose concentrations still fluctuated between 11 mmol/L and 22 mmol/L in the daytime, while hypoglycemia occurred at night. Subsequently, acarbose (100–100-50 mg three times a day) and sitagliptin (50 mg per day) were prescribed for symptom management, and his glucose concentrations returned to the normal range. Laboratory findings at a 1-year follow-up showed an HbA1c concentration of 7.0%, insulin concentrations of 138.53/733.40 pmol/L at 0/2 h, and C-peptide concentrations of 1.35/2.21 nmol/L at 0/2 h, respectively.

### Case 2

This patient was a 71-year-old man who was admitted because of polydipsia and polyuria that had persisted for 8 years, as well as recurrent episodes of unconsciousness throughout this period. He was diagnosed with type 2 diabetes mellitus 8 years before admission. Acarbose was initially prescribed, but was switched to insulin aspart 30 (8–12 units twice a day) because of poor glucose control 5 years previously. Three months previously, the dose of insulin aspart 30 was gradually reduced to 4–10 units because of hypoglycemia. However, he had unexpected episodes of unconsciousness due to hypoglycemia 12 days before admission. Moreover, his symptoms were obviously relieved after food intake. He was subsequently treated with voglibose instead of insulin injections. However, the hypoglycemic symptoms continued to appear between 02:00 am and 03:00 am, despite the absence of other treatments. A laboratory examination showed the following: C-peptide values were 15.22/17.59/19.73/15.22 nmol/L at 0/1/2/3 h, and insulin concentrations were > 2089.5/> 2089.5/> 2089.5/> 2089.5 pmol/L at 0/1/2/3 h during the OGTT, respectively. The anti-insulin antibody test was also positive. His treatment regimen was changed to prednisone (5 mg three times a day), acarbose (50 mg three times a day), metformin (0.5 g twice a day), and glargine (12–18 units twice a day) for glucose control. Insulin injections were also gradually discontinued. Following this treatment, he showed improved glucose control. Laboratory tests during a recent follow-up showed the following: C-peptide levels of 0.77/2.40 nmol/L at 0/2 h and insulin levels of 430.13/1350.21 pmol/L at 0/2 h during the OGTT, respectively.

### Case 3

This patient was a 79-year-old man who presented with a 3-year history of hyperglycemia and a 1-year history of polydipsia. At the time of presentation, he had been in a hypodynamic state for 15 days. Three years previously, he was diagnosed with impaired glucose tolerance without any symptoms, but no treatment was provided. One year earlier, he developed polydipsia, hyperuresis, and cutaneous pruritus, and lost weight. His random plasma glucose concentrations increased to as high as 20 mmol/L. Consequently, he was started on aspart 30 (21 units before breakfast and 8 units before dinner) and metformin (0.5 g twice a day). One month previously, he visited our hospital because of complaints of recurrent midnight hypoglycemia, particularly at approximately 02:00 am. During that period, although an insulin injection was gradually reduced and subsequently discontinued, his glucose concentration still dropped to 3 mmol/L. Laboratory tests showed that C-peptide and insulin concentrations were above the normal range, and the insulin autoantibody test was positive. At that time, non-classical IAS was considered as the probable cause of repeated hypoglycemia, and treatment was changed to acarbose (50 mg three times a day). After 15 days, blood tests showed the following: fasting plasma glucose level, 3.71 mmol/L; anti-insulin antibody, > 45.4 U/mL; and insulin concentrations were 1567.44/1493.06/1370.20/1616.43/1812.01 pmol/L at 0/30/60/120/180 min and C-peptide concentrations were 3.41/3.68/3.95/4.56/4.51 nmol/L at 0/30/60/120/180 min during the OGTT, respectively. For treatment of IAS, prednisone therapy was started at 12 mg daily (4 mg per dose three time daily) then reduced to 8 mg daily after 2 weeks and to 4 mg per night 1 week later. The diabetes treatment plan was changed to human biosynthetic insulin injection (16 units per day), sitagliptin (50 mg per day), and voglibose (0.3–0.2 mg twice a day). Repeated laboratory tests showed the following: C-peptide concentrations were 0.73/1.67 nmol/L at 0/2 h, respectively, and the insulin concentration was 728.14 pmol/L at 0 h during the OGTT. The glucose concentration fluctuated between 7 and 12.9 mmol/L.

### Case 4

This patient was a 52-year-old man diagnosed with type 2 diabetes mellitus at the local hospital according to a fasting glucose test that showed a concentration of 16 mmol/L. His postprandial glucose concentration was 27 mmol/L. However, the patient was asymptomatic. He was then treated with a 50/50 Mixture Recombinant Human Insulin Injection (Gansulin 50R) of 12–16 units twice a day, and the dose had been adjusted to 6–10 units 2 months previously. During this period, his glucose was well controlled. One month previously, he developed dizziness and was flustered again. He also started sweating excessively, and at that time, his fasting plasma glucose concentration was 2.8 mmol/L. One week previously, the treatment was changed to acarbose 0.1 mg three times a day because of repeated occurrence of hypoglycemic symptoms. His insulin concentrations were 1294.01/1303.77/1456.7/2057.11/2032.41 pmol/L at 0/30/60/120/180 min and C-peptide concentrations were 1.30/1.80/2.21/2.87/2.55 nmol/L at 0/30/60/120/180 min during the OGTT, respectively. The anti-insulin antibody test was positive. He was prescribed metformin (0.5 g twice a day), acarbose (0.05 g three times a day), aspart (6–5-5 units three times a day), and methylprednisolone (4 mg three times a day) for glucose control. After these changes, hypoglycemia syndrome did not recur (Table 1).

**Table 1:** The related information about the patient

Characteristic	Patient1	Patient2	Patient3	Patient4
History of DM (years)	14	11	4	3
Current Age	79	71	79	52
BMI	26.57 kg/m2	26.35 kg/m2	26.23 kg/m2	20.78 kg/m2
Original Treatment	Metformin	Acarbose	Nov 30:36–16; Metformin	Gansulin 50R: 16–12
Insulin (type and dose)	Insulin pump Lispro	Nov30 12–8 units BID	Nov 30 36–16 units BID	Gansulin 50R 16–12 units BID
Other drug used	n.a.	Amiodarone	Meloxicam; Diclofenac;	Benazepril Hydrochloride
Additional disease	Gout; Renal insufficiency	Hypertension; Coronary heart disease	Hypertension; Arthralgia	Hypertension; Rheumatic arthritis;
Autoantibodies (detected)	AIA(+) TG (+)35.47μg/L TGA(−)	AIA(+) ANA(−) dsDNA(−) Sm(−) RF(−) SSA(−) SSB(−)	AIA(+) TGA(+) 5.93 IU/mL↑ ANA(+) 1:40 dsDNA(−) Sm(−) RF(−) SSA(−) SSB(−)	ANA(+) 1:40 MPO-ANCA(+) 326.4 AU/mLCCP(+) 249.88 RU/mL RF (+)65.50 IU/ml↑ TG(+) 348.65 μg/L dsDNA(−) Sm(−) SSA(−) SSB(−)
Endocrine				
IGF-1	n.a.	199.7 ng/mL↓	1209.0 ng/mL↑	78.2 ng/ml -
GH	n.a.	0.4 ng/ml	0.75 ng/ml	0.4 ng/ml
TSH	5.36mIU/L	4.33miu/L	1.29miu/L	1.32miu/L
free T4	12.5 pmol/L	11.41 pmol/L	11.57 pmol/L	15.21 pmol/L
ACTH(8 am-4 pm-0 am)	32.06–39.16-47.01 pg/mL	30.6–20.4-49.2 pg/ml	41.6–16.5-9.9 pg/ml	29.5–10-16.9 pg/ml
GC(8 am-4 pm-0 am)	411–406-168 nmol/L	294.64–267.93-449.96 nmol/l	414.65–195.04-71.57 nmol/l	493.75–206.77-315.97 nmol/l
Pancreatic magnetic resonance	n.a.	Normal	Focal cystic change	n.a.

Abbreviations *n.a* not available, *IGF-1* insulin like growth factor receptor 1 *GH* growth hormone *TSH* thyroid stimulating hormone *T4* thyroxine, *ACTH* adrenocorticotropic hormone, *GC* glucocorticoid, *BID* twice a day, *AIA* anti-immunoglobulin antibodies, *TG* thyroglobulin, *TGA* anti-thyroid-globulin antibody, *ANA* anti-nuclear immune body, *dsDNA* anti-double stranded DNA antibody, *Sm* anti-Sm antibody, *RF* Human rheumatoid factor, *MPO* Human Mouse myeloperoxidase, *ANCA* anti-neutrophil cytoplasmic antibody, *CCP* anti-cyclic peptide containing citrulline, *SSA* Sjögren’s syndrome A, *SSB* Sjögren’s syndrome B

## Discussion and conclusions

We experienced four cases of IAS that all shared the following characteristics: [1] recurrent episodes of symptomatic hypoglycemia [2]; prior exposure to exogenous insulin; and [3] high concentrations of plasma insulin immunoreactive antibodies and hyperinsulinemia after discontinuing insulin injection. All four cases manifested symptoms, such as classical IAS. Therefore, we temporarily identified these cases as non-classical IAS. We review hyperinsulinemia-related diseases and discuss their distinct features compared with other cases below.

Many endocrine diseases can present with endogenous hyperinsulinemic hypoglycemia (EHH). EHH is diagnosed with the presence of inappropriately high serum insulin concentrations while plasma concentrations of glucose are < 55 mg/dL and ≤ 70 mg/dL in individuals with and without diabetes, respectively [4, 5]. IAS is a rare cause of EHH, characterized by spontaneous hypoglycemia, extremely high serum insulin levels (> 1000 pmol/L), and high titers of insulin autoantibodies against endogenous insulin [1]. After a meal or oral glucose load, increased glucose levels can stimulate insulin secretion, but autoantibodies bind to these insulin molecules, rendering them unavailable to exert their effects. The resulting hyperglycemia further promotes insulin release. The inappropriately increased concentrations of free insulin eventually cause hypoglycemia. Most patients with IAS achieve remission with nutritional management [6], and small frequent meals with low carbohydrates are preferred [7]. Additionally, glucocorticoids and immunosuppressants are prescribed to ameliorate immune dysregulation as well as avoid hypoglycemic attacks in IAS [8–12]. Other therapeutic options have also been shown to be successful in the management of IAS (e.g., acarbose for decreasing endogenous insulin secretion) [13]. In addition, plasmapheresis and rituximab can be used to eliminate insulin autoantibody titers in the circulation [1, 8, 12].

Aside from IAS, another type of autoimmune hyperinsulinemia is type B insulin resistance [2, 14]. The diagnosis of type B insulin resistance syndrome is based on the presence of antibodies directed against the cell surface insulin receptor [14–16]. These autoantibodies prevent endogenous insulin from binding to insulin receptors and decrease transduction of the insulin signal [17, 18]. Hyperinsulinemia in these patients has been attributed to increased secretion of insulin to compensate for peripheral insulin resistance and concomitant reduction in insulin clearance [17, 18]. Type B insulin resistance syndrome is characterized by severe hyperglycemia and is less common hypoglycemia compared with IAS [17, 18]. Treatment strategies for type B insulin resistance syndrome should be based on the specific requirements and individualized. Many of these patients undergo spontaneous remission of autoantibodies after 11–48 months of treatment with insulin and an insulin sensitizer. In severe cases, intravenous methylprednisolone and cyclophosphamide are recommended [14, 17].

Apart from autoimmune-induced EHH, insulinoma and nesidioblastosis should also be considered. Insulinoma is a small (< 2 cm) and benign tumor that is the most common neuroendocrine tumor of the pancreas [19, 20]. Secretion of endogenous insulin cannot be suppressed by hypoglycemia in insulinoma [21]. The common diagnostic criteria for insulinoma include increased concentration of insulin (≥43.05 pmol/L), C-peptide (≥0.2 nmol/L), and proinsulin (≥5 pmol/L). Surgical resection is the first-line treatment for insulinoma [22]. Nesidioblastosis is a condition involving diffuse hyperplasia of the pancreatic islets [23]. Formation of nesidioblastosis is attributed to congenital or acquired excessive function of abnormal pancreatic β-cells [24]. Additionally, subtotal pancreatectomy is a good option because of the diffuse nature of the disease [25] (Table 2). Furthermore, insulin receptor-negative insulin resistance also leads to hyperinsulinemia. Activation of the protein kinase Cu and phosphorylated insulin receptor substrate-1 Ser-307 by intramyocellular lipids can lead to muscle insulin resistance [26]. Moreover, activation of IκB kinase affects insulin signaling by phosphorylating and switching off the function of insulin receptor substrate, leading to increased insulin concentrations [27]. Hyperinsulinemia is also present in overtreatment with exogenous insulin. The difference between exogenous insulin-and endogenous insulin-induced hyperinsulinemia is the lowering of plasma glucose, lipoproteins, and inflammatory markers [28].

**Table 2:** Differential diagnosis of hyperinsulinemia

Subject	Insulin resistant syndrome	Type B insulin resistance	Insulinoma	Nesidioblastosis
Clinical manifestation	hyperandrogenism, widespread acanthosis nigricans, insulin resistance autoimmune disorders	hyperandrogenism, widespread acanthosis nigricans, insulin resistance autoimmune disorders	inappropriately high serum insulin concentrations during an episode of hypoglycemia	inappropriately high serum insulin concentrations and hypoglycemia
Mechanism	insulin autoantibodies	insulin receptor antibodies	neuroendocrine tumor	excessive function of abnormal pancreatic β-cells
Treatment	Steroid (Prednisone, 5-10 mg/day) immunosuppressan	insulin and insulin sensitizer steroid and immunosuppressant	complete resection	partial or subtotal pancreatectom.
Serum insulin levels	generally above 1000 pmol/L		≥43.05 pmol/L	↑(moderate)

In the current case series, each of the four patients was found to have non-classical IAS with high concentrations of anti-insulin antibodies that were caused by exogenous insulin injection. These antibodies can cause hypoglycemia, even after insulin injection is discontinued. In patients with classical IAS, hypoglycemia is typically postprandial. As glucose concentrations eventually fall, insulin secretion also subsides, and the total insulin level decreases. This leads to an increase of released free insulin concentration that is inappropriate for the glucose concentration, resulting in postabsorptive hypoglycemia [1]. However, most of our cases had fasting hypoglycemia, which were attributed to dissociation of insulin and insulin antibodies while the internal environment changed at night (which may be related to decreased affinity of the insulin antibody). Insulin antibodies bind with sufficient insulin in the daytime, accounting for less antibodies to be attached in the middle of the night. Therefore, increased free insulin concentrations could induce fasting hypoglycemia.

In the treatment of non-classical IAS, a suitable dose and disposition of insulin are required for better glycemic control. However, while case 3 was in a hypoglycemic status, the patient was treated with the same insulin and it was gradually discontinued, and in case 4, the type of insulin was changed. On the basis of our experience, we prefer to switch to another type of insulin if islet function appears to be poor. This is because changing to another insulin type can make the insulin antibodies disappear and glycemic concentrations more stable. However, if islet function is good, we prefer to gradually reduce the same insulin dose and add oral drugs. Although steroids can aggravate hyperglycemia, they are always used to treat IAS and non-classical IAS in clinical practice due to the following reasons. First, steroids reduce the hypoglycemia phenomenon that occurs in the middle of the night in non-classical IAS. Second, steroids decrease insulin antibodies and have advantages for glycemic control in the daytime. Therefore, we justify the treatment effectiveness in our patients through stability of glycemic concentrations.

However, discontinuing previous insulin treatment and switching to steroid treatment may worsen glucose control in patients with diabetes mellitus. Therefore, further studies are required to determine whether a smaller dose of prednisone in combination with a suitable dose of insulin can specifically treat non-classical IAS **(**Table 3**)**.

**Table 3:** The relationship between classic and non-classic insulin resistance

Subject	Classic IAS	Non-classic IAS
Clinical manifestation	Postprandial hypoglycemia	Fasting hypoglycemia
Diagnosis	high titers of autoantibodies against endogenous insulin, without exposure to exogenous insulin	high titers of autoantibodies against exogenous insulin exposure
Medical Treatment	Glucocorticoids (30-60 mg per day) and immunosuppressant	a small dose of prednisone (15 mg per day) in combination with a suitable dose of insulin
serum insulin levels	serum insulin concentration > 1000 pmol	serum insulin concentration > 1000 pmol

## Data Availability

Not applicable

## Abbreviations

EHH: Endogenous hyperinsulinemic hypoglycemia
IAS: Insulin autoimmune syndrome
OGTT: Oral glucose tolerance test
